# Structural and Functional Insights into α-Synuclein Fibril Polymorphism

**DOI:** 10.3390/biom11101419

**Published:** 2021-09-28

**Authors:** Surabhi Mehra, Laxmikant Gadhe, Riya Bera, Ajay Singh Sawner, Samir K. Maji

**Affiliations:** Department of Biosciences and Bioengineering, IIT Bombay, Powai, Mumbai 400076, India; laxmikantgadhe@gmail.com (L.G.); biome.bera@gmail.com (R.B.); ajaysinghsawner@gmail.com (A.S.S.)

**Keywords:** α-synuclein, amyloid, polymorphs, synucleinopathies

## Abstract

Abnormal accumulation of aggregated α-synuclein (α-Syn) is seen in a variety of neurodegenerative diseases, including Parkinson’s disease (PD), multiple system atrophy (MSA), dementia with Lewy body (DLB), Parkinson’s disease dementia (PDD), and even subsets of Alzheimer’s disease (AD) showing Lewy-body-like pathology. These synucleinopathies exhibit differences in their clinical and pathological representations, reminiscent of prion disorders. Emerging evidence suggests that α-Syn self-assembles and polymerizes into conformationally diverse polymorphs in vitro and in vivo, similar to prions. These α-Syn polymorphs arising from the same precursor protein may exhibit strain-specific biochemical properties and the ability to induce distinct pathological phenotypes upon their inoculation in animal models. In this review, we discuss clinical and pathological variability in synucleinopathies and several aspects of α-Syn fibril polymorphism, including the existence of high-resolution molecular structures and brain-derived strains. The current review sheds light on the recent advances in delineating the structure–pathogenic relationship of α-Syn and how diverse α-Syn molecular polymorphs contribute to the existing clinical heterogeneity in synucleinopathies.

## 1. Introduction

Misfolding and aggregation of α-synuclein (α-Syn) play a crucial role in the progression of neurodegenerative diseases, such as Parkinson’s disease (PD), dementia with Lewy bodies (DLB), Parkinson’s disease dementia (PDD), and multiple system atrophy (MSA), altogether termed as synucleinopathies [1]. As a result of protein aggregation, the neuropathological changes occur in the brain over time and the affected brain regions show α-Syn immunopositive inclusion bodies [2,3]. These inclusion bodies are defining characteristics of synucleinopathies and termed as Lewy bodies (LBs) and Lewy neurites (LNs) in PD, DLB, and PDD, and glial cytoplasmic inclusions (GCIs) in MSA [1,4]. LB formation has also been shown to be the major driver of neurodegeneration [5]. It involves an interplay of fibrillation, interactions with membranous components of the cell, and post-translational modifications (PTMs) [5,6,7]. Although inclusion bodies in synucleinopathies exhibit differences in size, shape, structure, and locations in the brain [1], the common trait is the presence of filamentous aggregates of α-Syn protein. These α-Syn filaments are highly ordered cross-β-sheet structures formed by the aggregation of the protein [8]. Numerous in vitro studies have demonstrated that α-Syn undergoes a structural transition from unordered state to cross-β-sheet-rich fibrils [9,10,11], which are similar to α-Syn amyloids found in the inclusion bodies in diseased patients’ brains [12,13,14,15,16]. However, the aggregation of α-Syn is not a single-state conversion process; instead, it involves the interconversion of multiple conformational states and the formation of oligomeric intermediates [17]. These oligomeric intermediates are highly heterogeneous, transient and metastable and suggested to be the most toxic species during the fibrillation pathway [17]. However, the transient and highly heterogeneous nature of these oligomeric species makes it challenging to explain their structure–toxicity relationship [17]. On the contrary, amyloid fibrils formed as the end-product of the aggregation process are primarily involved in the prion-like propagation of aggregates and contribute significantly to the spread of the disease pathology [18,19,20,21,22,23]. Nonetheless, there have been studies that have found it difficult to induce the α-Syn pathology in mice following intracerebral administration of fibrils [24], conceivably due to the size and dimension of full-length mature fibrils. Instead, several groups have suggested that non-fibrillar or oligomeric species are involved in the spread of α-Syn pathology [25,26,27]. However, considering the toxic nature of oligomers, it is difficult to establish the link between the toxicity and spread by the oligomers. Therefore, the molecular mechanism governing the interplay of oligomers and fibrils needs to be fully understood.

Further, the polymorphic nature of amyloid fibrils has added another complexity in the field. Detailed structural models of amyloid fibrils and aggregation intermediates have revealed that amyloid fibrils exhibit polymorphism at the molecular level, i.e., a single peptide or protein can form a range of distinct, self-propagating fibrillar assemblies [28]. Recent findings from biochemical and structural studies involving cell lines, animal models, and human brain extracts have provided initial evidence that structural variations in amyloid fibrils can be responsible for the observed disease variations [29,30,31,32,33,34,35]. The fibril growth conditions, such as buffer composition, salts, temperature, etc., profoundly affect the morphology and biological activity of α-Syn fibrils formed in vitro [29,31,33,36,37,38]. This suggests that change in the solution conditions alter the molecular interactions between the polypeptide chains, leading to different fibril types. Polymorphism can also be observed due to differences in the pattern of inter or intra-residue interactions, the number of amino acid residues constituting protofilaments, their packing, and orientation [39,40,41]. Several other factors are also responsible for the polymorphic nature of amyloids; however, our understanding of how a particular solution condition leads to the formation of different fibril structures is limited.

The existence of polymorphs has been linked to the strain phenomenon in prions, where different strains of PrP protein are associated with a range of clinical phenotypes observed in prion diseases [42,43,44]. In vitro, prion strains are characterized by differences in protease resistance, glycosylation profile, electrophoretic mobility, seeding ability, etc. In vivo, they are distinguished based on the clinical signs and symptoms, lesion profile, disease onset, and incubation period [45,46,47]. An increasing body of evidence suggests that α-Syn also exhibits prion-like strain phenomena, which explains its association with various neurodegenerative diseases with distinct clinical and pathological phenotypes [35,48,49,50]. Lee and co-workers showed the generation of synthetic strains of α-Syn capable of differentially cross-seeding tau for the first time [30]. This study formed the basis of later investigations involving α-Syn strains [29,32,33,36,49,51,52,53,54]. Recent advances in solid-state NMR (ssNMR) spectroscopy and cryo-electron microscopy (cryo-EM) have further contributed to understanding the molecular-level polymorphism in α-Syn fibrils [55,56,57,58,59]. These fibril polymorphs can be distinguished based on the fibril diameter, presence of twists, number and packing of protofilaments, side-chain interactions, the secondary and tertiary structure arrangement, etc. [41,57,60,61]. Cryo-EM structures of fibrils of wild-type (WT) α-Syn and its mutational variants have also provided novel insights into how disease-associated point mutants of α-Syn alter the fibril structure of the WT protein, suggesting polymorphism within the mutants. Further, characterizing and solving the structure of patient-derived strains can provide direct proof of the existence of α-Syn strains responsible for disease heterogeneity in synucleinopathies.

Overall, various previous studies have suggested that different strains of α-Syn are responsible for the clinical variations observed in synucleinopathies and possibly explain the association of α-Syn aggregates with disease heterogeneity [32,33,34,35,52,53]. Yet, there are questions about the origin of polymorphism in vivo, propagation of strain-specific properties of fibrils, and factors governing the formation of strains that need attention. The present review focuses on the polymorphic nature of α-Syn and describes its role in the disease pathogenesis of PD and related disorders. It discusses the evidence demonstrating that α-Syn can assemble into distinct fibril strains and could be the primary drivers for the disease heterogeneity in synucleinopathies.

## 2. Clinical and Pathological Features of Synucleinopathies

The aggregation of α-Syn protein is associated with PD and other neurodegenerative disorders, collectively termed as synucleinopathies. These include MSA, DLB, PDD, and less characterized neuroaxonal dystrophies [4,48]. PD is the most common among all synucleinopathies and has been a prime focus of α-Syn research conducted over the decades. α-Syn misfolds and accumulates in the form of fibrillar inclusion bodies in synucleinopathies. However, the appearance and location of these inclusion bodies vary in different synucleinopathies [62]. For instance, neuronal inclusions are present in PD, PDD, and DLB, whereas glial inclusions are formed in MSA [4] and less characterized axonal spheroids in neuro-axonal dystrophies [4]. The existence of diverse clinical and pathological profiles in synucleinopathies raises a fundamental question of how the aggregation of a single protein leads to different diseases.

### 2.1. Parkinson’s Disease (PD)

PD is the second most widespread and complex neurological disorder after Alzheimer’s disease (AD) [63]. It is a prevalent, chronic, and progressive neurodegenerative disorder, affecting approximately 1% to 4% of the general population over 60 and 80 years of age [64,65,66]. It involves the accumulation of eosinophilic, round, cytoplasmic LBs and LNs, accompanied by the degeneration of dopaminergic neurons in the substantia nigra pars compacta (SNpc) region of the midbrain [3,67]. The loss of dopaminergic neurons results in a decrease in the level of neurotransmitter dopamine [68], which results in abnormal brain functioning and impairment in motor functioning that leads to PD symptoms. Four cardinal symptoms, such as bradykinesia, resting tremor, postural instability, and rigidity are considered for clinical diagnosis of PD [67]. In addition to these motor symptoms, non-motor symptoms like insomnia, constipation, cognitive dysfunction, autonomic failure, and depression are also observed in PD patients [67]. Several studies claim that these non-motor symptoms and many gastrointestinal (GI) tract problems [69,70,71,72,73] in patients involve the enteric nervous system (ENS) affected in the early stages of PD. Experimental data suggest that misfolded α-Syn spreads in a prion-like fashion from ENS to CNS through innervations of the dorsal motor nucleus of the vagus nerve (DMV) [74,75,76]. However, the factors that cause α-Syn to misfold and aggregate in ENS are not fully known. One of the reported factors is the high prevalence of Enterobacteriaceae within the GI tract that produce extracellular amyloids termed curli fibers [77]. These curli amyloids are used for host attachment, tissue invasion, and biofilm production by bacteria. Despite the functional roles of curli fibers, curli-producing *Escherichia coli* induces GI dysfunction and motor impairment in mice overexpressing α-Syn [78]. The amyloidogenic subunit of curli fibrils (CsgA) interacts and accelerates the aggregation of α-Syn and curli expression is indeed required to induce α-Syn associated behavioral deficits [78]. However, further research is needed to decipher the role of bacterial amyloids in promoting α-Syn aggregation and tracing the origin of PD along the gut-to brain axis.

In the past few years, the genetics of PD have been studied markedly. The SNCA gene has been identified as one of the major genes linked to sporadic and familial PD [63,79]. Mutations in parkin and LRRK2 are the other common genes associated with recessively and dominantly inherited PD, respectively [63,79]. The duplication [80] and triplication [81] of the SNCA gene, the gene encoding for α-Syn, causes early onset of parkinsonism. Along with the multiplication of the SNCA gene [82], single point mutations are also associated with familial autosomal parkinsonism. To date, seven missense mutations are known to be associated with familial PD: A30P [83], E46K [84], H50Q [85,86], G51D [87], A53T [88], A53E [89], and the newly discovered A53V [90]. Aggregation and amyloid formation of these familial mutants have been extensively studied in vitro [12,16,91,92,93,94,95,96,97,98,99,100,101]. A30P, A53E, and G51D slow down the aggregation of WT α-Syn, whereas E46K, A53T, H50Q, and A53V accelerate the same [10,91,92,93,94,95,99,101,102]. However, their aggregation rate in vitro does not correlate with the disease onset, suggesting an interplay of oligomerization and fibrillation in vivo, which dictates the disease progression and onset in familial forms of PD [103]. Apart from genetic factors, ~95% cases of PD are sporadic [63,104] and are associated with cellular and environmental risk factors. These include the presence of polyamines, chaperons, glycosaminoglycans, membranes, metal ions, exposure to pesticides, and heavy metals, etc. [6,105,106,107,108,109,110,111,112,113,114]. These risk factors uniquely modulate the misfolding and aggregation of α-Syn associated with PD pathogenesis [94,115].

### 2.2. Multiple System Atrophy (MSA)

MSA is a rare sporadic neurodegenerative disease and becomes progressively chronic with an autonomic failure along with symptoms of parkinsonism or cerebellar ataxia [116,117]. The prevalence of the disease is 2.4–4.9 cases per 100,000 population [117]. MSA affects both genders equally, and the incidence is more prevalent in people above 60 years of age [118,119]. MSA is a more devastating and aggressive neurological disorder than other synucleinopathies because of more rapid clinical progression with much shorter disease duration (6–9 years) than PD (~12 years) [117,119]. Many patients diagnosed with PD are actually found to have MSA after autopsy [120]. This misdiagnosis of MSA happens due to overlapping symptoms of the two disorders, suggesting that the prevalence of MSA is more than the estimation [117]. MSA was previously described by three clinical syndromes, striatonigral degeneration, olivopontocerebellar atrophy, and Shy-Drager syndrome, formerly thought of as separate disorders [121,122]. Later, it was found that these syndromes often coexist clinically and pathologically and give the impression of a common underlying disease, which was termed as MSA. Clinically, MSA patients display numerous combinations of symptoms like parkinsonism, cerebellar ataxia, progressive autonomic failure, and pyramidal signs. Based on that, they are categorized into two main clinical subtypes: (i) the parkinsonian subtype (MSA-P), with parkinsonism as a predominant feature, and (ii) the cerebellar subtype (MSA-C), with cerebellar ataxia as a major trait [123,124,125]. The occurrence of MSA-P and MSA-C ranges from 2:1 to 4:1, respectively [126,127,128]. However, the MSA-C subtype is majorly found and predominates in the Japanese population [118].

The histopathological hallmark of MSA is the presence of GCIs formed in the oligodendrocytes in the brain, which show strong immunoreactivity with α-Syn [129]. This makes it pathologically distinct from other synucleinopathies as it shows an abnormal accumulation of α-Syn protein in oligodendrocytes, unlike PD and DLB, where α-Syn inclusions are found in neurons [130]. Although some MSA patients have shown the presence of α-Syn aggregates in the nucleus and cytoplasm of neurons [131], these neuronal inclusions are less prevalent than GCIs in MSA. Furthermore, the mechanism of aberrant accumulation of α-Syn in glial cells is unclear, as there is no or minimal expression of α-Syn in mature oligodendrocytes [132,133,134,135]. A few reports have suggested the possibility of transmission/translocation of α-Syn from neurons to oligodendrocytes [136,137]. However, this mechanism is not completely known, and the exact origin of α-Syn inclusions in oligodendrocytes remains obscure.

### 2.3. Dementia with Lewy Bodies (DLB)

DLB is the second most common α-synucleinopathy after PD [138,139,140]. The incidence and prevalence rates of DLB are not accurate because of overlapping symptoms with AD, PDD, vascular dementia, and other synucleinopathies. It is estimated that its prevalence is approximately 0.4%, i.e., 400 people per 100,000 population in the elderly [141], accounting for 5% of all dementia cases and between 1–4 people per 1000 population [142]. Initially, DLB was identified as dementia syndrome [143]. Later, the inclusion bodies from DLB patients were found to be highly immunoreactive to α-Syn [3]. After that, it was categorized as one of the main types of synucleinopathies. While PD is characterized by a decline in motor abilities, DLB is mainly characterized by dementia. Instead, a DLB patient may or may not suffer from parkinsonism [144]. Unlike PD, the LBs in DLB patients are mainly localized and distributed in the cytoplasm of cortical neurons of the diseased brain [145]. Cortical LBs are eosinophilic, rounded, and generally lack the halo structure seen in classical LBs. Clinically, it is characterized by dementia, memory impairment, parkinsonism, and changes in behavior, sleep, and autonomic bodily and cognitive functions [143].

Many DLB patients also show significant Aβ deposition in the cortical area, along with the formation of LBs [146,147]. Several compelling pieces of evidence support the synergistic relationship between Aβ and α-Syn [148,149,150,151,152,153,154]. In vitro studies have demonstrated that α-Syn and Aβ can cross-seed, form hetero-oligomers, and promote the aggregation of each other [148,151,152,154]. Consequently, shorter disease duration and more rapid decline have been observed in patients with AD pathology and dementia [155,156]. A recent study provided direct experimental evidence of the effect of co-pathology where Aβ plaques promoted the seeding and spreading of α-Syn in mice with abundant Aβ pathology [157]. Still, clinical and pathological studies suggest that DLB overlaps more with PDD than AD [158].

Overall, the synucleinopathies are associated with abnormal deposition of α-Syn but still vary in terms of clinical and pathological phenotypes. Despite several studies, the reason for this clinicopathological divergence remains a puzzle.

## 3. Misfolding and Aggregation of α-Syn

**Figure 1:**
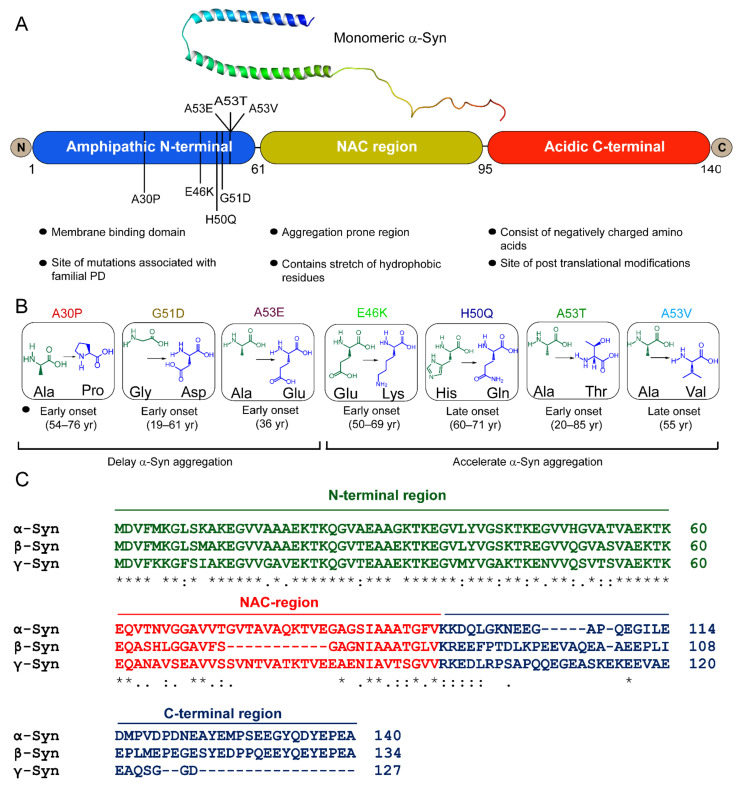
α-Syn structure and its disease-associated mutations. (**A**) Structure of micelle-bound human α-Syn [PDB ID: IXQ8] [167] and schematic representation of the primary sequence of α-Syn with three distinct domains, (i) the N-terminus (blue) contains lipid-binding motif and is the site of all familial mutations of α-Syn, and (ii) the central NAC domain (mustard) contains the stretch of hydrophobic residues. The two curved α-helices, helix-N (Val3-Val37) (blue) and helix-C (Lys45-Thr92) (green) in the micelle-bound α-Syn [1XQ8], connected by a short linker are formed within the 11 aa repeats (consensus sequence), which extends up to the first 89 residues [167]. (iii) the C-terminal (red) is rich in acidic amino acids. (**B**) Schematic diagram of seven mutational variants of α-Syn associated with familial PD along with their age of onset. A30P, G51D, and A53E delay and E46K, H50Q, A53T/V accelerate α-Syn amyloid formation. (**C**) Multiple sequence alignment of α-Syn, β-Syn, and γ-Syn by Clustal W. “*” indicates identical amino acids in all three variants, “:” and “.” indicate conserved and semi-conserved residues, respectively.

Monomeric α-Syn is an intrinsically disordered protein and tends to adopt multiple conformational states affected by solution conditions like pH, temperature, ionic strength, viscosity, etc. [9]. For instance, the presence of alcohols (ethanol) or fluoroalcohols (TFE or HFiP) induces the formation of β-sheet or α-helical partially folded structures of α-Syn, depending on the concentration and the type of alcohol used [9]. α-Syn was first isolated from the antisera raised against the cholinergic vesicle from Torpedo californica, an electric ray [159]. Due to its location at the nuclear envelope and presynaptic terminal, it was named synuclein [159,160]. α-Syn protein is encoded by the *SNCA* gene mapped to the human chromosome 4q21.3-q22 [160]. α-Syn was also discovered by Ueda et al. [161] during the study of amyloid plaques from the brains of patients with Alzheimer’s, in which they identified a non-amyloid-β component (NAC) in the plaques, which was derived from a precursor protein, NACP [161]. It was detected in all the tissues except the liver, and the highest concentration was found in the brain [161]. Later, it was found that NACP is a natively unstructured and human homolog of α-Syn [162,163,164]. Extensive biophysical and structural characterization revealed that α-Syn is a 140 amino acid protein with a molecular weight of ~14.4 kDa and pKa of 4.7 [165]. It is known to be involved in neurotransmitter release, vesicle trafficking, and SNARE complex assembly in the brain, though its exact physiological role is still obscure [160,165]. α-Syn consists of three domains, N-terminal, NAC, and C-terminal domains (Figure 1A). N-terminal of α-Syn (residues 1–60) is an amphipathic, lysine-rich, and lipid-binding domain, which interacts with the membranes [109]. It contains 11 aa repeats, including conserved KTKEGV hexameric motifs [109]. These repeats are conserved across species as well as among three synuclein members. Although α-Syn remains unordered in an aqueous solution, it adopts a helical structure involving N-terminus upon association with negatively charged small unilamellar vesicles or detergent micelles [109,166,167]. Interestingly, all the familial mutations of α-Syn also occur in the N-terminus region [83,84,85,86,87,88,89,90] (Figure 1B). The NAC domain of α-Syn (residues 61–95) forms the protein’s hydrophobic core and is prone to aggregation. This domain is responsible for the conversion of α-Syn from an unordered state to β-sheet-rich fibrils [168,169]. NAC is also part of the membrane-binding domain of the protein [167]. The conformational ensemble of α-Syn monomer indeed consists of structures that are similar to the membrane-bound state of α-Syn [170]. These contain partially folded helices involving N-terminuses and NAC domains similar to the 1XQ8 model [170], suggesting that such a type of folding might also be present in the early stages of aggregation. The C-terminal domain (residues 95–140) is flexible and predominantly consists of negatively charged amino acids [165]. The disordered carboxy-terminal part is also involved in the nuclear localization of α-Syn protein and its interaction with metal, small molecules, and proteins [171,172,173,174,175].

β-Synuclein (β-syn) and γ-synuclein (γ-Syn) proteins also belong to the synuclein family [165] (Figure 1C). β-Syn is 134 amino acid protein, earlier identified as the human homolog of bovine phosphoneuroprotein 14 (PNP14). The 11-amino-acid (residues 73–83) stretch is missing in its NAC domain (Figure 1C), due to which it lacks the ability to fibrillate [11,176,177]. Earlier, it was believed that β-Syn is an inhibitor of α-Syn aggregation and prevented its neurotoxicity [11,176]. However, this notion changed after discovering missense mutations in β-Syn gene, P123H (familial), and V70M (sporadic), known to cause DLB [178]. The deleterious effects of these mutations have been shown by cell- and animal-based studies [179,180]. Our group recently showed that under normal physiological conditions, fibrilization and aggregation of β-Syn and its disease-associated mutations did not occur, but an altered microenvironment, such as a decrease in pH and/or presence of heparin, caused them to polymerize [181]. γ-Syn, which shares ~55% sequence homology with α-Syn, was initially identified in breast cancer malignancies encoded by a breast-cancer-specific gene, *BCSG1* [182]. It was reported in the peripheral central nervous system and breast cancer tissues [182,183]. It aggregates and forms fibrils in vitro [177] and in cells [184], but is comparatively slower than α-Syn [91].

**Figure 2:**
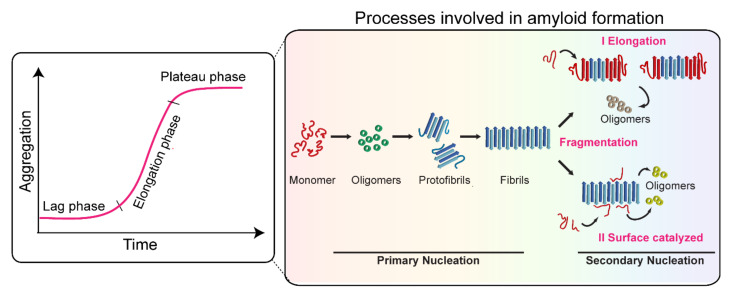
Processes involved in α-Syn aggregation. The amyloid growth kinetics of α-Syn follow three different phases, (i) the lag phase, (ii) the elongation phase, and (iii) the stationary/plateau phase. Primary nucleation, secondary nucleation, fragmentation, and elongation processes are active through all the phases of the growth curve, however, at different rates.

The misfolding and fibrillation of α-Syn is a major event in several neurodegenerative disorders [185]. The misfolded α-Syn aggregates are amyloidogenic in nature, which aberrantly accumulate in the brain, and, as a result, the patient suffers movement abnormalities that worsen over time. The aggregation of α-Syn is a complex phenomenon and involves the conversion of monomers to highly ordered cross-β-sheet-rich structures through the formation of several soluble on- and off-pathway oligomeric species [185,186]. The amyloid formation of α-Syn is generally monitored by thioflavin T fluorescence dye [187]. It follows sigmoidal growth kinetics, which consist of (i) the lag phase, involving the formation of nuclei, which eventually grow into the detectable aggregate structure in solution; (ii) the elongation phase, the conversion and subsequent growth of oligomeric species into the fibrillar structure; and (iii) the stationary phase, representing the steady-state where the monomer and fibril concentration reaches the equilibrium. By the end of the aggregation, α-Syn assembles into atypical long amyloid fibrils, normally characterized by electron microscopy and atomic force microscopic imaging techniques [9]. These phases of aggregation cannot be attributed to a single event or microscopic process. Instead, all the processes, viz., primary nucleation, elongation, secondary nucleation, and fragmentation, are active through all the phases of the growth curve but at different rates [188,189] (Figure 2). These reaction rates are governed by aggregation rate constants and the concentration of the reacting species at a given time [190]. The amyloid formation initiates with primary nucleation of the monomeric species in the solution and elongation by addition of monomer to growing ends of the aggregates [189]. However, primary nucleation processes are short-lived and rapidly surpassed by secondary nucleation processes [191]. The fragmentation of the fibrils under agitation conditions (or even under quiescent conditions depending upon the stability of amyloid fibrils) modifies the number of growing ends and significantly affects the overall growth kinetics [188,189]. Moreover, secondary nucleation by surface catalysis is also one of the major contributors to amyloid growth in several systems, especially under quiescent conditions [189,190].

The self-assembly and aggregation of α-Syn is a complex phenomenon and involves multiple parallel processes. Therefore, it is crucial to understand the underlying molecular events to delineate their fundamental connection with human disease.

## 4. Prion-like Strain Phenomena in α-Syn

Since the discovery of α-Syn as the main constituent of Lewy body pathology in 1997, the primary focus has been shifted in delineating the underlying pathogenic mechanism of PD. Heiko Braak [192] presented a staging system of Lewy pathology in 2003 based on the specific patterns of α-Syn spreading. According to the Braak hypothesis, the Lewy pathology initiates from the olfactory bulb and DMV and then progressively spreads to the other brain regions. Although there is experimental and clinical evidence supporting Braak’s hypothesis, it is uncertain whether it is applicable and/or accurately describes the progression of PD in all the patients. For instance, there are cases in which patients do not show Lewy pathology in DMV or ENS, while other brain regions are severely affected [193,194,195,196,197,198]. Even in some cases, no link has been observed between the severity of Lewy pathology and clinical symptoms in PD [195]. Therefore, it is suggested to only apply Braak’s hypothesis to a subset of the population [198] as not all PD patients adhere to the staging system proposed by Braak [199]. Intriguingly, the reports of Lewy pathology in fetal neuronal grafts after fourteen years of transplantation into the striatum of the PD patient provided direct proof of cell to cell transmission and the spreading of α-Syn pathology proposed by Braak [200,201]. Studies using in vitro and cell model systems later suggested that α-Syn aggregates are infectious, can move from one cell to another, and seed the aggregation of their soluble endogenous counterpart in the recipient cells, explaining the phenomenon observed in grafted neurons [18,21,23]. This prion-like transmission of α-Syn aggregate from one region to another is also implicated in DLB and PDD patients, suggesting that the spread of Lewy pathology is the shared property of α-Syn aggregates in synucleinopathies [202,203,204,205]. However, the clinical and pathological features of these synucleinopathies are highly variable and heterogeneous [206,207], [146,208]. One might ask, why, despite being linked to the aggregation of the same protein, the distribution of α-Syn pathology and the manifestation of disease symptoms are different amongst synucleinopathies. This could be explained by the prion-like strain phenomenon of α-Syn, in which the same precursor protein forms different fibrils that result in distinct pathology.

### 4.1. Concept of Prion Strains

The last few decades of research have suggested that proteins/peptides with various structures and sequences can form a common fold of cross-β-sheet-rich structure of amyloid [209,210,211,212,213]. These proteins/peptides form amyloids with a common aggregation framework, i.e., through nucleation-dependent polymerization mechanism [214,215]. However, each protein/peptide may also undergo a distinct aggregation pathway to form a unique amyloid structure. Recent high-resolution structural studies with ssNMR and cryo-EM have indeed suggested that each protein packs uniquely and forms different structures for the cross-β-sheet fold [56,57,58,61,216]. Not only that, but, surprisingly, one protein can form multiple different structural folds [40,59]. Thus, these proteins can adopt various conformations from the same amino acid sequence, giving rise to several proteinopathies and, therefore, not confirming the one protein–one structure hypothesis [217]. For instance, tau folds differently in Alzheimer’s and Pick’s disease [40,218,219]. Different TAR DNA-binding protein (TDP-43) aggregates exist in the brains of Frontotemporal lobar degeneration (FTLD-TDP) subtypes, showing morphological differences across the subtypes [220]. This protein’s ability to misfold and display conformational diversity can lead to severe consequences, such as neurodegeneration [221]. This phenomenon of a protein to form different amyloids associated with various phenotypic properties is well known for prions [46,222]. Prions are infectious protein particles that show conformational heterogeneity and can be transmitted from one individual to another [223]. A myriad of evidence shows epidemiological and clinicopathological diversity in human prion diseases, such as Kuru disease, Gerstmann–Straussler–Scheinker syndrome, and Creutzfeldt–Jakob disease [224], as well as non-human prion diseases, such as bovine spongiform encephalopathy (BSE) in cattle, scrapie in sheep and goats, etc. [225]. The normal cellular prion protein (PrP^C^) undergoes conversion from α-helical to β-sheet-rich conformation (PrP^Sc^), which is an insoluble, PK-resistant, and infectious form. PrP^Sc^ propagates and aggregates following two widely accepted mechanisms/models, i.e., the template-assisted and nucleation polymerization model. A pathogenic prion acts as a template in the template-assisted model and provides a surface for converting an endogenous normal prion protein to its misfolded pathogenic form [226]. In the nucleation–polymerization model, monomeric PrP^Sc^ combine and form a stable nucleus, also called a seed. These seeds keep on recruiting PrP^C^ and convert them to their pathogenic counterparts [226]. One of the remarkable properties of prions is that they can misfold into diverse conformations, each giving rise to distinct clinical, histological, and pathological profiles. These aggregates with different conformations and pathological behavior are referred to as ‘strains’ [45,227]. The pioneer observations on the presence of prion strains came from the study by Pattison and Milson, 1961 [228], wherein they experimentally produced scrapie in goats and observed distinct clinical manifestations of the disease owing to different strains. In another study, Fraser and Dickinson were able to distinguish different strains of scrapie in infected mice models depending upon the extent of damage in different regions of the brain [229]. Later on, a plethora of reports showed the existence of PrP^Sc^ strains [227,230,231,232,233] and methods to distinguish them, such as Proteinase K (PK) digestion [234,235], electron paramagnetic resonance (EPR), and NMR spectroscopy [43]. These conformations of PrP^Sc^ vary in terms of different types of secondary structure elements, such as α-helix, β-strand, β-turn, or different structural folds or different packing [227,230,232,235,236]. For instance, prion protein from the Syrian hamster refolds into both α-helical and β-sheet structures, as well as various intermediates in aqueous solution [236]. These are not only structurally but also functionally distinct from each other [43] and have been identified in different human and animal prion disorders [233,237].

Despite resulting from the aggregation of the same prion protein, the prion diseases differ from each other with respect to the disease onset/incubation period, progression, and histopathological lesions in the infected brain [224,225]. This has been collectively termed as ‘prion strain phenomena’ [45,46,238]. One of the major contributors of this strain diversity/variation is the ability of the infectious agents/prions to infect certain species [44,239,240], and cross the ‘species barrier’, thereby generating a variety of strains with distinguishable biochemical and pathological characteristics. One such case is vCJD (variant Creutzfeldt–Jakob disease), resulting from the interspecies transmission of BSE prion from cattle to humans [44,240], which is the only known case of non-human prions being transmitted to humans. Also, different prion strains have been known to coexist with each other, as seen in the case of sCJD (sporadic Creutzfeldt–Jakob disease) [241,242], showing distinct biochemical properties in different regions of the brain. Prion strains are also known to extend their incubation period upon co-infection with other strains, thus showing a phenomenon of ‘competition’, as observed in various studies [243,244,245]. All the above-mentioned features of prion strain phenomena and the potential for the generation of new prion strains have emerged not only as a serious scientific challenge, but also as a threat to general public health. Recent studies, however, have suggested that this strain property of amyloids is not only limited to prions but other amyloids associated with various neurodegenerative disorders such as Alzheimer’s and Parkinson’s [19,246,247], [18,248]. Various evidence has been provided from in vitro and in vivo studies to demonstrate the prion-like strain behavior of α-Syn, as discussed below.

### 4.2. α-Syn Strains Generated In Vitro

**Figure 3:**
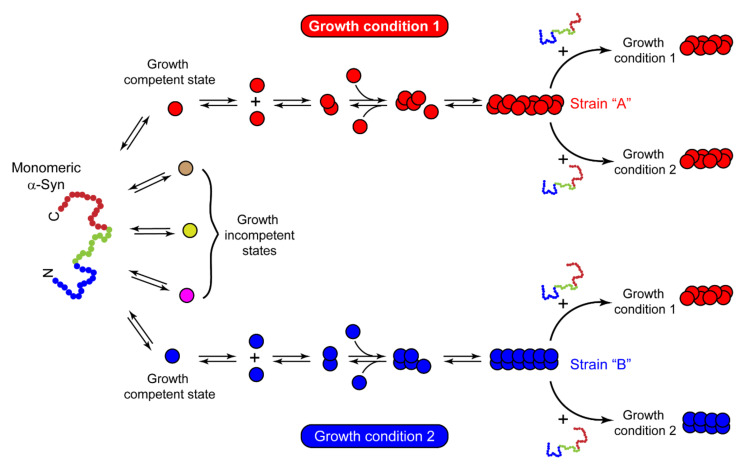
Mechanism of strain formation and its propagation. Natively unstructured monomeric α-Syn populates into multiple conformational states with distinct abilities to form amyloid fibrils. Red and blue circles represent the growth-competent states, which, under certain sets of growth conditions 1 and 2, form different fibril strains ‘A’ and ‘B’, respectively. Strain ‘A’ seeds the monomeric protein and passes on its structural architecture to the next generation of fibrils, irrespective of growth condition 1 or 2. In contrast, strain ‘B’ passes its structural characteristics on the daughter fibrils only in the same assembly conditions that were used for its growth (growth condition 2). This suggests that the nature of seeds and growth conditions play an essential role in deciding the fate of assemblies upon cross-seeding. The other conformations in brown, yellow, and pink circles cannot yield thermodynamically stable intermolecular interactions and are incapable of growth. These are referred to as growth-incompetent states.

Growing evidence of prion-like strains of α-Syn associated with clinical and pathological variations observed in synucleinopathies has been reported in the past few years [29,30,31,32,33,34,35,249]. These strains have been defined as distinct and stable conformational assemblies of a single protein that can self-replicate and propagate in vivo and result in different disease phenotypes. Guo et al. discovered two ‘strains’ of α-Syn pre-formed fibrils (pffs), through de novo fibrilization, termed as ‘strain A’, and repetitive seeding fibrilization in vitro, termed as ‘strain B’. These two strains exhibited distinct conformations and striking differences in cross-seeding tau protein in primary neuronal cultures and in vivo [30]. Besides documenting the evidence of α-Syn fibril strains for the first time, these findings also identified the cross-seeding behavior of α-Syn. Later, several groups took advantage of the chameleon property of α-Syn [9] and screened numerous growth conditions to generate α-Syn strains in vitro. Under different growth conditions, several conformationally stable and unstable states of protein were observed [250]. The conformations, which are not thermodynamically stable or cannot establish stable intermolecular interactions, cannot grow into amyloid fibrils. These are referred to as growth-incompetent states (Figure 3). On the other hand, growth-competent states of α-Syn can grow and form different fibrils depending on the growth conditions (Figure 3). The resulting fibrils under different assembly conditions not only possess different biochemical and biophysical properties, like resistance to proteases, cytotoxicity, seeding ability, etc. [29,30,31,32,34,35,36,51,53], but also imprint their architecture on the daughter fibrils depending on the growth condition and the nature of seeds (Figure 3). Bousset et al. indeed generated two structurally and functionally different α-Syn strains, named ‘fibrils’ and ‘ribbons’, using different physiological salt concentrations [29]. Fibrils caused more cytotoxicity, whereas the ribbons were found to be more effective in inducing α-Syn inclusions in vivo [33]. Similar differences were obtained with strains generated by Suzuki et al. [51], where one strain caused the accumulation of abundant phosphorylated and ubiquitinated α-Syn aggregates in cultured neurons and mice due to its ability to interact with proteasome complexes, whereas the other strain failed to do so [51]. Nowadays, varying experimental conditions have rather become a common strategy to generate strains in vitro. In cases where the criteria to be called a ‘strain’, i.e., should be a structural variant of the protein aggregates, exhibit the ability to self-propagate and serially transmit the disease over the next generations and cause clinical and phenotypical disease variations, are not completely fulfilled, it would be more appropriate to call the fibrils as ‘polymorphs.’ These polymorphs may show striking differences in their morphology and structure [28,56,251], nucleation rates [252], seeding and membrane binding ability in cells [36], etc. However, as a functional consequence of these structural variations in polymorphs, they may or may not result in distinct clinical subtypes of diseases. There are also cases of intrasample polymorphism, which can arise irrespective of whether fibrils are generated in vitro [39,57,253,254,255,256] or derived from brain extracts [40,257,258]. While these may possess certain commonalities, like a similar monomeric fold or share a common structure, they also show marked differences in morphology, β-strand arrangement, or biochemical properties [57,256]. Thus, different assembly conditions can generate structurally and functionally distinct fibrillar assemblies, which may either propagate as a unique α-Syn strain or may simply form polymorphs.

Several reports have claimed the presence of α-Syn aggregates in the gastrointestinal tract [259,260,261,262,263] and a key role of the vagal nerve in spreading these aggregates from the gut to the brain [74,76,192,264,265]. Recently, curli-expressing *E. coli* has been shown to promote α-Syn pathology in the gut and the brain of mice overexpressing human α-Syn [78]. It is possible that exposure to microbial amyloids induces polymorphism. Instead, it would be interesting to ask if different forms of α-Syn (polymorphs) originate from the gut, spread to the brain regions via retrograde vagal transport, and cause pathology in a strain-specific manner. In this context, structural and functional differences have been observed between α-Syn fibrils formed in the presence and absence of bacterial endotoxin lipopolysaccharide (LPS) [31]. LPS is known to modulate α-Syn aggregation by stabilizing the α-helical intermediates formed during its aggregation pathway, resulting in the fibrils with variable cytotoxicity and altered internalization behavior [266].

Furthermore, α-Syn has been shown to undergo numerous PTMs, like phosphorylation, methionine oxidation, acetylation, nitration, etc., which are directly associated with its aggregation and cytotoxicity [94,115,267,268]. As pS129 is the most common PTM and the main form of α-Syn in the inclusion bodies, it may cause strain formation in α-Syn in vivo. Ma et al. showed that phosphorylation at Ser129 enables the protein to form a distinct strain that differs structurally with higher cytotoxicity and different propagation properties in vitro and in cells compared to the wild-type counterpart (without phosphorylation) [269]. Not only phosphorylation, but also N-terminal acetylation may impart fibril polymorphism [267], suggesting that even a slight modification in α-Syn amino acid sequence can significantly impact its fibril structure. The alteration in the fibril structure may further influence the propagation of fibrils in vivo by selectively targeting distinct cell types and cellular populations within the brain [32]. These strain-specific differences are then faithfully preserved over the generations [28,29,52] and lead to the clinical differences in disease onset, neurological illness, lesion profile, etc. [32]

Overall, these studies suggest that in a complex and crowded milieu such as that of a cell, subtle environmental and cellular changes, the presence of co-factors or other proteins, and alterations in the protein’s primary sequence may lead to the formation of different polymorphs or strains, resulting in different disease outcomes.

### 4.3. α-Syn Strains in Human Synucleinopathy Samples

The structural and functional differences observed in recombinant strains can be validated by identifying and characterizing fibrils directly from synucleinopathy patient samples. The first evidence of a brain-derived strain came from a seminal study by Prusiner et al. [270], which demonstrated that brain extracts from MSA are transmissible to transgenic mice and cells, resulting in abundant α-Syn pathology [270]. In contrast, this was not observed using brain extracts from PD, suggesting that the PD-derived strain may differ from MSA [270]. Then comes the question, what might lead α-Syn to adopt a different conformation in MSA or PD? In vitro, various solution conditions (like the presence or absence of salt) give rise to fibrils with different structural and functional properties [29]. Similarly, α-Syn is also exposed to several microenvironments in vivo, affecting its aggregation [271]. The dopaminergic neurons in PD and the oligodendrocytes affected in MSA belong to different cell lineages and have distinct cellular environments. Lee and co-workers demonstrated that distinct intracellular environments of two cell lines impart strain formation in MSA and PD [34]. α-Syn fibrils derived from GCIs in oligodendrocytes (GCI-α-Syn) and LBs in neurons (LB-α-Syn) of diseased brains differ significantly and exhibit distinct seeding abilities [34]. GCI-α-Syn strain is highly effective in seeding α-Syn aggregation compared to LB-α-Syn, thereby contributing to the aggressiveness of MSA [34].

α-Syn aggregates have also been detected in biological fluids like cerebrospinal fluid (CSF) and plasma of PD patients [272,273]. α-Syn aggregation begins years before the onset of actual disease symptoms and, thus, the detection of these aggregates at early stages may enable the identification and characterization of a particular strain in these fluids. In this context, the amplification of α-Syn aggregates from brain extracts of PD and MSA patients using the protein misfolding cyclic amplification (PMCA) technique has been recently developed. This technique involves the amplification of misfolded proteins in vitro, in a manner similar to DNA amplification by PCR [274]. It consists of alternate cycles of incubation and sonication, resulting in amyloid replication. First, the trace amount of amyloid is incubated with an excess of native protein to induce polymer growth. Then, the sample mixture is subjected to sonication, which will break down the fibrils, resulting in several nuclei. Each newly formed nucleus will then act as a seed in the next cycle and further induce the growth of fibrils. This way, after each cycle, the number of seeds will increase exponentially and will allow the detection of the minute amount of misfolded aggregates present at the beginning [274]. Soto and co-workers used PMCA to amplify the α-Syn aggregates from the CSF of the patients diagnosed with PD and MSA [52]. They found that PD- and MSA-derived fibrils exhibit different biophysical and biochemical properties and correspond to distinct conformational strains of α-Syn [52]. Even α-Syn aggregates amplified from PD and MSA brain homogenates have been shown to exert variable toxicity and neurodegeneration in human dopaminergic neurons, reflecting different disease severity observed in PD and MSA patients due to different strains of α-Syn [275]. These findings conclusively suggest that synucleinopathies can be distinguished based on the type of α-Syn strain present in the brain. However, the complexity in detecting aggregates arises when patient-to-patient heterogeneity is observed in the same disease. This heterogeneity in α-Syn aggregates amplified from PD patients’ brain extracts is greater than MSA brain extracts [276]. Strohaker et al. reported that the fibrils derived from PD and MSA do not exhibit markedly distinct structural properties [276], in contrast to findings reported by Soto and co-workers [52]. The possible reason for the contrasting observations could be the differences in the PMCA protocols used by the two groups [277]. Additionally, Strohaker et al. used a much smaller sample size than Soto’s group [52,276]. Other factors, like the genetic background of the patients, age of the selected patients, a load of α-Syn aggregates in different patients, presence of other components in the extracts, and region of the brain from where extraction was done, could also be responsible for these differences [276]. Similar contradictions also exist in the field of AD pathology. Recent findings on brain-derived tau samples have suggested patient-to-patient heterogeneity in the tau fibril conformations exist within the same disease, AD [278]. However, Goedert and colleagues observed the same type of tau conformation in all AD cases analyzed so far, suggesting that tau fibrils from a single disease (like AD or Pick’s disease) adopt a common structural fold [218,219]. Although the reasons and factors that drive this structural specificity in tauopathies are unclear, it could be due to multiple isoforms of tau, PTMs, interactions with other protein molecules, co-factors, etc. Recently, Scheres and Godert presented a hierarchal classification of tau fibrils from different tauopathies based on the folds of their filaments [279]. Whether a similar classification exists for α-Syn fibrils isolated from synucleinopathy samples remains to be determined. Recently, a great effort has been made to solve the structure of α-Syn derived from the human brain by Schweighauser et al., using Cryo-EM [280]. The group found that α-Syn filaments from the brain of DLB patients do not twist and are thinner than those derived from the brain of MSA patients [280], consistent with the previous findings [3]. The lack of twists in fibrils derived from DLB precluded the determination of 3D structure by cryo-EM and the differences in α-Syn fibrils derived from MSA and DLB patients were drawn based on two-dimensional class averaging [280]. Although we need more high-resolution structures derived from synucleinopathy patients to reach a definite conclusion, the present reports certainly strengthen the claims on the existence of distinct fibril types of α-Syn. Moreover, the structures of α-Syn filaments from PD cases are not yet available, but solving them in the future can significantly help to understand the disease mechanism and generate therapeutic approaches against synucleinopathies.

## 5. High-Resolution Structural Models of Existing α-Syn Fibril Strains

Various biophysical techniques have been used so far, like ssNMR, micro-electron diffraction, EPR, circular dichroism (CD), hydrogen/deuterium exchange NMR (HDX-NMR), and cryo-EM, to determine the structure of α-Syn fibrils at different resolutions. These techniques have laid the foundation of molecular-level polymorphism in fibrils. The β-sheet structure of the fibril core using ssNMR revealed two fibrils, form A and form B, by sequential assignment of 48 residues of the core [56]. The study elucidated the presence of two fibril polymorphs that may have formed due to different mechanisms for fibrillation [56]. Likewise, the two contrasting fibril structures, ‘ribbons’ and ‘fibrils’, generated in vitro showed differences in the length, distribution, and number of β-sheet elements in their fibril structure analyzed by ssNMR [29]. However, despite several attempts, how α-Syn fibril polymorphs differ in atomic structure has remained largely unknown. The revolution in structural polymorphism came after the structure of α-Syn fibril was solved using cryo-EM at the atomic level resolution. Stahlberg and the group revealed that the α-Syn fibrils (residues 1–121) consist of two identical protofilaments [61]. The β-sheets from each protofilament interact and stabilize the structure via hydrophobic zipper geometry [61]. Notably, the residues 50–57 located at the protofilament interface are also the site of familial PD mutants (A53T/V/E), H50Q, and G51D [61]. In this regard, it was predicted that these mutations might change the fibrillar structure, resulting in different fibril types. Subsequent cryo-EM studies of the structure of full-length α-Syn have shown the difference in chirality and a helical twist [281] compared to the C-terminal truncated α-Syn fibril structure (residues 1–121) [61]. It was believed that these differences in the structures of full-length (1–140) and C-terminally truncated fibrils could be due to fibril polymorphism. The direct proof of these theories was obtained by recent seminal studies that used cryo-EM to delineate the models of α-Syn fibril polymorphs. Li et al. identified two fibril polymorphs, ‘rod’ and ‘twister’, with a common protofilament kernel structure but different inter-protofilament interfaces [57]. Twister polymorphs display an ordered bent-β-arch motif whereas rod polymorphs recruit some additional residues to form a ‘Greek-key’ motif, as reported by other groups as well [55,61,281]. The existence of polymorphs in rod and twister forms suggests that differences in the packing of the same kernel structure can lead to polymorphism. Similar observations have also been made for other amyloid proteins, like β-amyloid and tau, where the protofilaments with the same kernel structure but different packing arrangements lead to polymorphic structures [40,282]. Further, the two new polymorphic forms of α-Syn fibrils generated in vitro, named polymorphs 2a and 2b, respectively, are different from previously reported polymorphs 1a and 1b [57,61,281]. In polymorph 1a [61], the interactions between the residues at the protofilament interface are mediated by the formation of hydrophobic steric-zipper geometry, whereas in polymorphs 2a and 2b, they are mediated by salt bridges [41,61]. The closer inspection of the structural differences between polymorphs 1a/1b and new polymorphs 2a/2b revealed further differences in the arrangement of the β-arch motifs, which change the interface of the protofilaments between polymorphs 1 and 2 [41]. These studies strengthen the hypothesis that the same precursor protein α-Syn can assemble into multiple fibril polymorphs in vitro, which radically differ from each other in terms of atomic resolution.

Goedert and his colleagues recently studied cryo-EM structures of tau fibrils derived from Alzheimer’s and Pick’s disease patients’ brains [40,218,219]. They found distinct folds of tau filaments in both diseases, indicating different conformers of tau exist in different tauopathies. The same group reported two types of α-Syn filaments, type I and type II, from the brains of individuals suffering from MSA [280]. They found that each filament is made up of two non-identical protofilaments. The cavity formed by the close packing of the protofilaments encloses additional molecules that are yet to be determined [280]. The 2D class averaging also revealed different fibrils from MSA and DLB patients, suggesting the existence of distinct conformers associated with synucleinopathy. Further, it would be interesting to ask whether the patient-derived fibrils exhibit any level of similarity with the fibrils generated in vitro. Molecular-level characterization and comparison of brain-derived fibril samples with fibrils generated in vitro revealed that these two are structurally different [276,280]. The major difference between the MSA-derived and synthetic filaments is the size and packing of the protofilaments in MSA fibrils [280]. Moreover, researchers use harsh conditions to generate and isolate synthetic fibrils like agitation, salt concentration, etc., which affect the packing and β-sheet arrangement of filaments [10,283,284,285]. As a result, it becomes difficult to correlate the in vitro results with in vivo scenarios. On the other hand, one may only get pre-formed fibrils from disease-extracted samples but may not understand how they have originated and what factors have governed the formation of different fibrils in different brain samples. It is challenging to isolate the transient toxic species or intermediates from the brain samples to understand the disease pathogenesis. Consequently, we need to rely on in vitro samples to delineate the mechanisms of fibril formation, pathways, and kinetics analysis. Similarly, we have to test to the same using brain-derived fibrils to develop a more extensive knowledge base.

Overall, the high-resolution structures of α-Syn polymorphs could aid researchers in their quest for potential therapeutic targets. However, a thorough investigation is required to understand the impact of cellular conditions, mutations, PTMs, presence of co-factors, etc., on α-Syn fibril structure and the link of different fibril polymorphs with the clinical variability observed in PD.

## 6. Familial Mutations of α-Syn Form Distinct Fibril Conformations

α-Syn oligomerization and aggregation are associated with PD pathogenesis. Seven familial missense mutations have been discovered so far in the *SNCA* gene, associated with early- and late-onset PD [83,84,85,86,87,88,89,90]. Among these PD-associated mutations, E46K, H50Q, A53T, and newly discovered A53V mutants accelerate the rate of α-Syn aggregation, whereas A30P, G51D, and A53E mutations slow down the aggregation kinetics in vitro [91,93,96]. However, the link between the rate of aggregation (in vitro) and the age of the disease onset (in vivo) is not straightforward [103]. Although oligomers formed during the early stages of aggregation kinetics are potentially toxic [286], only A30P shows faster oligomerization and delayed conversion of oligomers into fibrils [102]. G51D, on the other hand, exhibits slow oligomerization and slow fibril formation [287,288], yet is associated with the early onset of disease. Due to this complexity in the behavior of familial mutants, it is challenging to set up a unifying mechanism by which they cause the disease.

Previous reports have suggested the α-Syn adopts a helical structure upon binding with membranes in vivo [289,290]. Any single amino acid change in the N-terminus domain of α-Syn may alter the membrane-binding ability and increase the cytosolic concentration of the protein by promoting faster aggregation [291,292]. The membrane-binding data of familial α-Syn mutants from our laboratory [92] and others [110,288,292,293] have shown that H50Q, A53T, and E46K mutants exhibit increased membrane binding, while A53E, G51D, and A30P mutants exhibit decreased membrane binding. This suggests that, similarly to aggregation, the membrane-binding capability does not correlate with increased disease propensities by familial α-Syn mutations. Therefore, there is a lack of correlation between the aggregation and membrane-binding ability with the actual disease pathogenesis caused by the familial mutants of α-Syn. This raises the question of how a point mutation in a natively unstructured protein shows drastic differences in the disease onset and progression. We believe it could be possible that different α-Syn mutants produce different types and amounts of oligomers and also may uniquely alter the seeding capacity of wild-type protein [103]. That is why mutants affect not only the overall aggregation rate of the protein, but also the microscopic steps involved in the amyloid formation, i.e., initiation and amplification of α-Syn through secondary nucleation process [294]. Intriguingly, Lazaro et al. found that, despite having identical oligomerization propensity in cultured cells, A30P, E46K, H50Q, G51D, and A53T exhibit distinct abilities to form inclusions [295]. A30P showed a decreased propensity to form inclusions in cells, whereas the E46K and G51D mutant displayed an opposite effect [295]. Again, the inclusion formation in cells [295] did not correlate with the aggregation propensity of mutants in vitro [12,91,92,93,102]. Thus, addressing these questions about how wild-type α-Syn and its mutants contribute to the early and late onset of PD becomes important to understand the differential pathogenesis of synucleinopathies.

**Figure 4:**
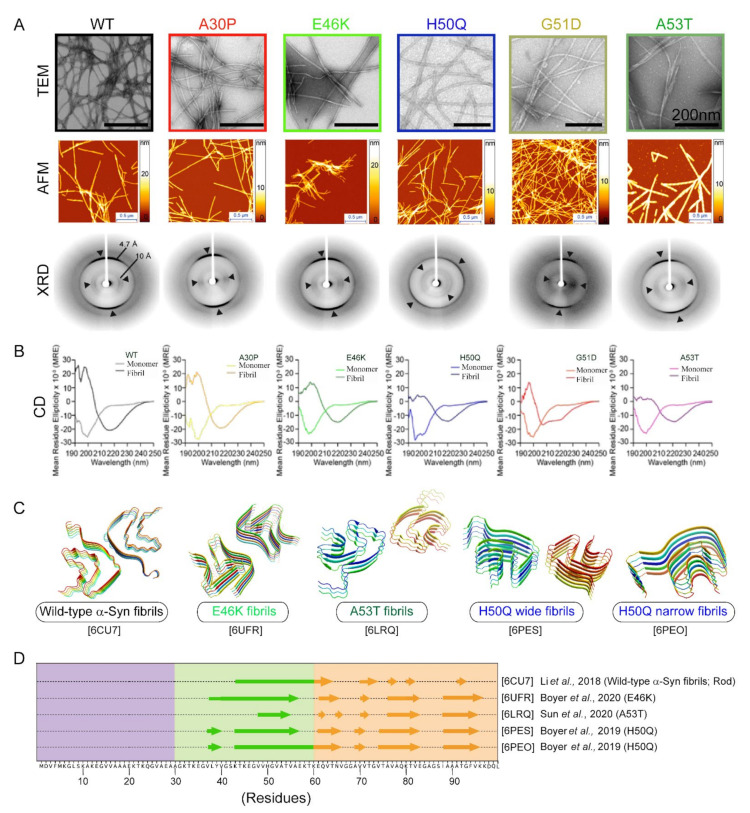
Polymorphism in WT α-Syn and its mutational variants. (**A**) *Upper and middle panel*. TEM and AFM images of WT α-Syn and its familial mutants (A30P, E46K, H50Q, G51D, and A53T) showing different fibril morphologies. *Lower panel*. X-ray diffraction pattern of fibrils formed by WT α-Syn and its point mutants showing a typical meridional reflection at 4.7 Å and variable equatorial reflection at ~8–10 Å among the wild type and mutants. (**B**) Secondary structure determination of WT α-Syn and its familial mutations by CD spectroscopy in its monomeric (light color) and fibrillar state (dark color). (**C**) cryo-EM models of WT α-Syn and its disease-associated mutants (E46K, A53T, and H50Q) fibrils. Corresponding PDB IDs of the structure are mentioned in the square bracket. (**D**) The schematic of 1–100 amino acids of α-Syn showing a comparison of β-sheet secondary structure in the WT and mutant fibrils. The α-Syn sequence is color-coded with residues 1–30, purple; 30–60, green; 60–100 orange to distinguish between the position of β-sheet elements in the different structures. *The TEM images for WT, A30P, and E46K are adapted with permission from D. Ghosh, P. K. Singh, S. Sahay, N. N. Jha, R. S. Jacob, S. Sen, A. Kumar, R. Riek and S. K. Maji (2015), Structure-based aggregation studies reveal the presence of helix-rich intermediate during α-Synuclein aggregation, Sci. Rep. 5:9228. Copyright © 2015, Macmillan Publishers Limited http://creativecommons.org/licenses/by/4.0/. H50Q and G51D TEM images are reprinted with permission from G.M. Mohite, S. Dwivedi, S. Das, R. Kumar, S. Paluri, S. Mehra, N. Ruhela, S Arunima, N. N. Jha, and S. K. Maji, ACS Chemical Neuroscience 2018 9 (11), 2628–2638. Copyright© 2018 American Chemical Society. TEM images of A53T mutants are reprinted with permission from G. M. Mohite, R. Kumar, R. Panigrahi, A. Navalkar, N. Singh, D. Datta, S. Mehra, S. Ray, L. G. Gadhe, S. Das, N. Singh, D. Chatterjee, A. Kumar, and S. K. Maji. Biochemistry 2018, 57, 35, 5183–5187. Copyright© 2018 American Chemical Society. AFM, XRD, and CD images reprinted with permission from F. S. Ruggeri, P. Flagmeier, J. R. Kumita, G. Meisl, D. Y. Chirgadze, M. N. Bongiovanni, T.P. J. Knowles, and C. M. Dobson ACS Nano 2020, 14, 5, 5213–5222. Copyright @2020 American Chemical Society. The atomic structures of fibrils are adopted from the given PDB IDs [57,298,299,300] and rendered using the molecular visualization system, PyMOL*.

Fibril formation is highly sensitive to changes in the local and/or global microenvironment of the protein. This suggests that a single amino acid change can result in polymorphism due to different site-specific conformational dynamics, as shown for the wild type and fibrils of E46K, A30P, and A53T [296]. In this context, Knowles and co-workers recently studied the systematic comparison of α-Syn and its disease-associated mutants using biophysical techniques [297]. PD mutants generate fibril polymorphs with distinct morphology and secondary structures compared to the wild-type protein [297]. Indeed, several reports have independently confirmed that different α-Syn mutants form fibrils with characteristic morphology revealed by transmission electron microscopy (TEM) and atomic force microscopy (AFM) studies, unique X-ray diffraction (XRD) patterns, and differences in secondary structure elements (Figure 4A,B).

Furthermore, the interface of the two protofilaments in the α-Syn fibril structure is formed by residues 50–57, which also harbors three familial mutations [61]. This suggests that even a single-point mutation can alter the dynamics and packing of the protofilaments. A closer inspection of the fibrils formed by mutants by cryo-EM [298,299,300] unveiled the plasticity of such fibrils in terms of twists, the number of interacting protofilaments, packing arrangement, secondary structure elements, and quaternary shape, etc. (Figure 4C,D). Boyer and his group studied H50Q mutant and found narrow (1c) and wide fibrils (1d) with one or two protofilaments, respectively [300]. Despite sharing the same conserved kernel structure as reported previously for wild-type α-Syn, the mutant fibrils displayed a new protofilament arrangement and hydrogen-bond networks [300]. Further, A53 lies in the center of the interface of the interacting protofilaments in wild-type α-Syn [61] and is also a hot spot for many point mutations [16,89,90]. Cryo-EM studies with N-terminally acetylated A53T mutant revealed no change in the fold of wild-type α-Syn [299]. However, the mutation disrupts the residue interactions and re-arranges the orientation of the protofilament interface, thereby resulting in a different type of fibrils [299]. This could also be the case with the other two A53 mutations, i.e., A53E and A53V, but this possibility is yet to be discovered. Cryo-EM modelling of fibrils formed by E46K mutation has also supported the prevailing hypothesis. It revealed the formation of fibril polymorphs with distinct protofilament packing and interfaces compared to wild-type α-Syn [298,301]. Besides, it formed a more stable and pathogenic variant of wild-type α-Syn [298]. Overall, these studies suggest that protofilament packing and the interface are critical in determining fibril structure. Each familial mutation may behave as a strain of α-Syn, uniquely altering the structure and dynamics of the resulting fibrils. These differences in the fibril structure may lead to different clinical and pathological outcomes, thereby contributing to disease heterogeneity in synucleinopathies.

## 7. Phase Separation and Nucleation: Molecular Basis of Fibril Polymorphism

Soon after the discovery that α-Syn aggregation is not only linked to PD but also MSA and DLB, the key research area has been primarily focused on understanding the ability of a protein to cause clinically and pathologically diverse neurodegenerative disorders. Over the past few years, the prion strain hypothesis has emerged as the leading explanation for the observed disease variability in synucleinopathies. Although a range of biophysical and biological data support the existence of α-Syn strains [29,30,32,33,34,35,53], it is still unclear how these strains originate and the factors that drive their formation in vivo. The probable answer could lie in delineating the aggregation pathways and discerning the molecular drivers underlying strain formation.

Amyloid formation is not only governed by primary nucleation, but is in fact dominated by secondary nucleation events through most of the aggregation growth phase, as discussed earlier [189]. This secondary nucleation involves the elongation of new fibrils from the existing seeds by recruiting monomers from the solution. Any changes in the solution conditions of the fibrils tend to alter the rate of primary and secondary nucleation events [188,189]. For example, acidic pH enhances the binding of α-Syn monomers to the fibril surface, thereby increasing the secondary nucleation rates [189,302]. Since seed-induced propagation of aggregates is attributed to the fragmentation and elongation of fibril seeds, this leads us to hypothesize that the strain phenomenon to be, at least in part, a consequence of secondary nucleation. Moreover, multiple species coexist at different stages of the aggregation kinetics [17,303,304], suggesting the involvement of aggregation intermediates in dictating the polymorphism. The mapping of the conformational space of α-Syn monomer reveals a structural subpopulation of α-Syn monomer that promotes its binding with membranes and induces the formation of various oligomers and fibrils [170]. Our lab recently demonstrated that the heterogeneous nucleation during the aggregation pathway forms the basis of the origin of polymorphism [252]. We generated two different polymorphs, HMFs (Helix matured fibrils) and PMFs (Pre-matured fibrils), from the aggregation intermediates formed under the same assembly conditions. PMFs do not have a stable amyloid core and possess random coil content along with β-sheet elements. Moreover, the structured β-sheet from the residues 74–79 is absent from its NAC domain (residues 65–80), suggesting PMFs to be less ordered fibril types. On the contrary, morphologically and structurally distinct HMFs are more compact and well-ordered with a stable fibril core [252]. These contain highly exposed hydrophobic surfaces and are potentially more toxic than less ordered PMFs. These polymorphs display not only structural differences but also exhibit different biological activities [252]. A similar study involving the identification of the aggregation intermediates in the presence of phospholipid membrane revealed that prefibrillar species contain two loop regions with residues 57 to 61 and 71 to 80 [305]. These intermediates then rearrange to species, which are fibrillar in nature, with most of the NAC region and the N-terminus (residues 38–80) forming the part of final fibril conformation [305]. Studies have also been reported with aggregates of Aβ, PrP, and tau, where distinct biological properties emanate due to structural differences between the polymorphs [28,306,307,308,309]. For instance, morphologically and structurally different Aβ fibrils formed under quiescent and agitating conditions show significant differences in toxicity in primary neurons, with quiescent fibrils being more toxic compared to other one [28]. A range of structurally diverse PrP^Sc^ conformations exhibits host cell tropism, with a specific set of strains preferentially targeting neurons, astrocytes, or even both [307,308].

**Figure 5:**
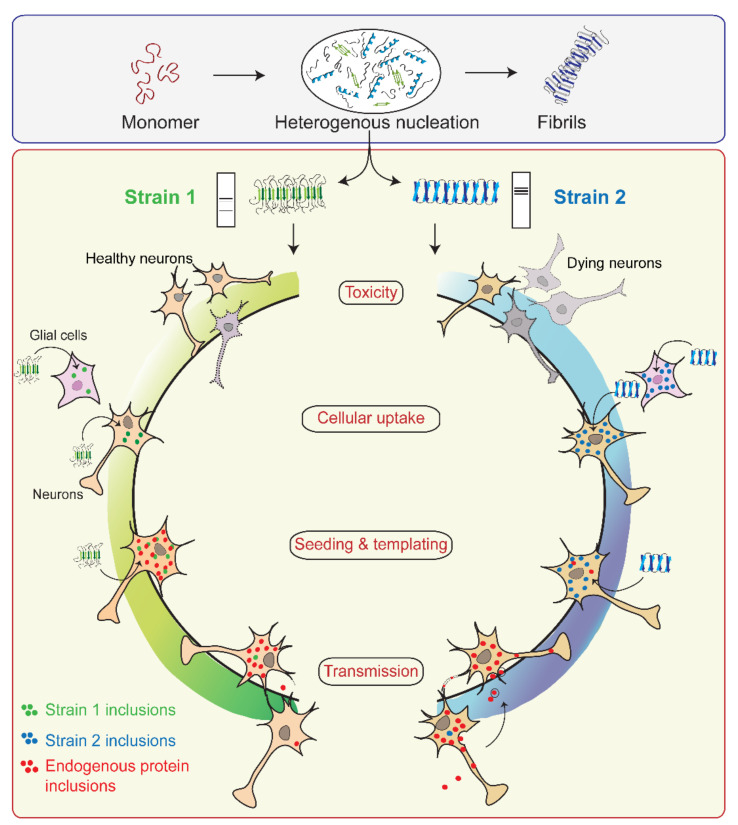
Prion-like behavior of α-Syn strains. Heterogeneous nucleation in the α-Syn aggregation pathway can lead to structurally and functionally distinct strains 1 and 2. These strains can exhibit variable cytotoxicity and prion-like properties of fibrils, which include cellular uptake, seeding, and templating and intracellular transmission of aggregates in cells. (*The figure is reproduced and modified from thesis entitled “Structural and Functional Insights into α-Synuclein Fibril Polymorphism: Implications in Synucleinopathies” by Surabhi Mehra (2020) IIT Bombay*).

Thus, α-Syn strains resulting from the heterogeneous nucleation in the aggregation pathway may cause clinical and pathological variations in PD by exhibiting variable cytotoxicity and different prion-like properties (Figure 5).

Recently, the evolution of the concept of protein aggregation to a more fundamental phenomenon, namely liquid–liquid phase separation (LLPS), has significantly influenced the field and directed the research in a new direction [310,311,312,313,314]. Proteins are incubated in varying conditions, for example, pH, temperature, and salt conditions [315,316,317]. The phase separation events are promoted in the presence of molecular crowders, like polyethylene glycol, dextran or ficoll, which aid in increasing the local protein concentration and facilitating the droplet formation [315,316,317,318]. Thereafter, the dynamicity, maturation and aggregation profile are investigated using a unique combination of biophysical, biochemical, and spectroscopic methods [315,317].

**Figure 6:**
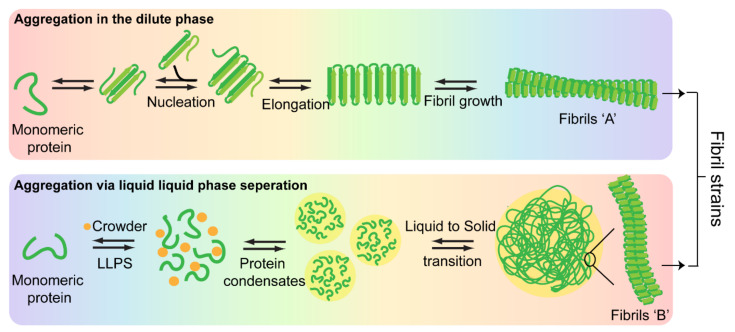
Strain formation under phase-separating and non-phase-separating conditions. Aggregation in the dilute phase involves primary and secondary nucleation events [189], with subsequent growth of nucleating seeds by the addition of monomers to the growing ends or via surface catalysis and maturation into fibrils ‘A’. Alternatively, fibril formation may also occur by liquid–liquid phase separation of the protein into dense liquid condensates facilitated by an increase in the local concentration of proteins in the presence of a crowder [325,326,327,328]. This phenomenon results in liquid to solid transition, eventually forming a hydrogel-like state consisting of fibrils ‘B’. Fibrils ‘A’ and fibrils ‘B’, resulting from the aggregation of the same precursor protein but formed under phase separating and non-phase separating conditions, can be different and may represent fibrils strains.

The phenomena of phase separation of various amyloidogenic proteins have been shown to precede aggregation and fibril formation. It has been suggested that the presence of intrinsic disorder regions (IDR’s), prion-like domains (PLD), and low complexity domains (LCD) promote the formation of phase-separated condensates of the amyloidogenic proteins [310,315,316,319,320]. Our lab recently demonstrated that the LLPS of α-Syn is a critical event in the early lag phase and precedes its aggregation under phase-separating conditions (presence of crowders, stressors, amyloid co-factors, etc.) [315]. The appearance of these phase-separated droplets in the lag phase of aggregation kinetics suggests that LLPS might enhance the nucleation events by increasing the local concentration of the protein molecules [315,320]. Moreover, the phase-separated α-Syn droplets undergo liquid-to-solid transition with time and result in the formation of amyloid hydrogel [315]. These amyloid hydrogels have been previously shown to entrap cytotoxic oligomers and fibrils [321], indicating the possibility that fibrils formed via LLPS could be toxic. However, under normal assembly conditions (without phase separation), α-Syn fibrils show very little or no cytotoxicity [322,323]. This suggests that the fibrils formed under phase separating and non-phase-separating conditions could be different. Moreover, the fibril formation in dilute solutions (known to occur via primary and secondary nucleation) [189] and that within the condensates (via LLPS) are not mutually exclusive events [324], but different aggregation pathways can result in the formation of different fibrils (Figure 6).

**Figure 7:**
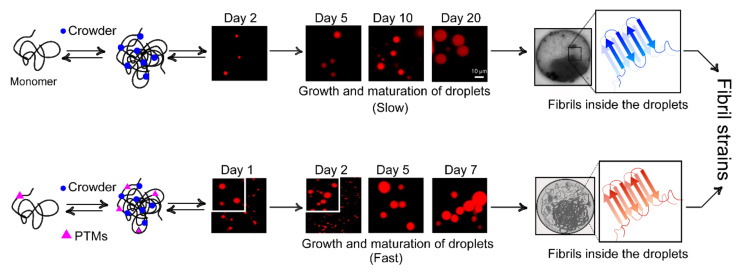
Formation of fibril strains under different phase-separating conditions. *Upper panel* shows the formation of labeled α-Syn droplets at day 2 and the subsequent transition of droplets from liquid to solid state over 20 days in the presence of molecular crowder PEG. Fibrils (illustrated in red) can be seen inside the droplet on day 20. *Lower panel* shows faster formation of S129E (a phosphomimetic mutant) α-Syn droplets at day 1. These droplets are bigger than that of the wild type and show faster growth and maturation (fibrils illustrated in blue). Fibrils arising under different LLPS conditions, like the presence of familial mutations, PTMs, small molecules, or metal ions can show polymorphism and represent distinct fibril strains. *The fluorescence microscopy images and the TEM image of the droplets are adapted with permission from Soumik Ray et al., α-Synuclein aggregation nucleates through liquid–liquid phase separation, Nature Chemistry, (2020) Copyright @2020 Springer Nature*.

For instance, TDP-43, a protein involved in ALS/FTD, undergoes fibrillation with or without LLPS. Still, the fibrillation kinetics in both cases are different, suggesting the involvement of complex processes in fibrillation in the presence of LLPS [329]. Therefore, it would be highly relevant to ask a few questions like whether the fibrils formed with or without undergoing phase separation are structurally and functionally distinct from each other, or, simply put, show fibril polymorphism? Whether different LLPS conditions or mutations have any role in deciding the strain behavior of the α-Syn fibrils formed? For example, the pathogenic mutations in FUS have been shown to exhibit different biophysical properties compared to the wild-type protein [327]. These findings suggest that, similarly to non-phase-separating conditions where different solution conditions give rise to polymorphs [29], fibrils formed under the different phase-separating conditions could also be different (Figure 7). For instance, α-Syn phase separating under different conditions, like the presence of PTMs, familial mutations, small molecules, or metal ions, can show polymorphism and form different types of fibrils (Figure 7). However, this phenomenon is yet to be determined as it would need extensive characterization of fibrils formed inside the droplets.

We believe that the co-existence of conformationally distinct intermediate species and a multitude of aggregation pathways observed with or without LLPS could form the basis of the origin of polymorphism. However, more research in this field is required to delineate the contribution of heterogeneous nucleation and multiple aggregation pathways to fibril polymorphism. The development and application of various biophysical techniques could help us gain insightful information about the molecular events occurring during LLPS and subsequent droplet maturation.

## 8. Clinical and Therapeutic Implications of Polymorphism

The studies discussed in the review provide conclusive evidence that α-Syn can form diverse polymorphs with distinct structural and biological properties. Similar to prions, where each prion disease is encoded by a distinct conformation of the misfolded protein, each synucleinopathy is also possibly associated with a unique α-Syn structure. However, diverse fibril structures for the same protein pose several challenges in drug development against neurodegenerative disorders. Therapies like immunotherapy would be highly specific for a particular strain but might fail to recognize a different one. Similarly, developing small therapeutic molecules or drugs for blocking or slowing down the protein aggregation process needs to be screened for multiple conformations. Considering the complexity of fibrils and its polymorphism, targeting monomeric α-Syn could be an option. However, that is also challenging due to the intrinsically disordered nature of the protein. Despite these challenges, detecting and characterizing patients’ derived α-Syn strains will open a window of opportunities for deeper understanding and characterization of synucleinopathies. It will facilitate the development of new therapies and more robust classification systems of synucleinopathies. In this context, highly sensitive techniques like PMCA [330], real-time quaking-induced conversion assay (RT-QuIC) [331] and HANdai amyloid burst inducer (HANABI) [332] have significantly contributed to amplifying α-Syn aggregates from CSF of patients’ brains. PMCA and RT-QuIC have been used to discriminate between PD/MSA and PD/DLB patient-derived α-Syn strains, respectively [52,333]. Employing these techniques would aid in monitoring the disease progression over time and help in the early and specific diagnosis of synucleinopathies.

Furthermore, the clinical and pathological differences and patient-to-patient heterogeneity observed in PD and related disorders may impact how synucleinopathy patients should be treated. As of now, all PD patients receive the same type of treatment, and no distinction is made between the patients depending on how they have acquired the disease and what symptoms they present. Therefore, we anticipate that drugs and therapeutic agents designed to target pathogenic α-Syn species may involve using either a single conformational-based drug or cocktail of drugs against diverse polymorphs or strains that are populated in the diseased human brain. Furthermore, several key parameters should be considered on a case-by-case basis, like disease profile, pathological and clinical symptoms, types and nature of the protein strains involved, and disease progression rate while treating patients with synucleinopathies. Therefore, identifying potential therapeutic targets and designing conformational-based drugs is the next big step towards developing drugs against PD and related disorders. Establishing the link between the propagation of a strain and the disease phenotype will provide valuable insights on developing effective strategies for combating neurodegenerative disorders. Another challenge is delivering drugs in the brain to treat neurodegenerative disorders, mainly facing setbacks due to restrictive blood–brain barriers [32]. Numerous attempts have been made to deliver drugs through nanocarriers, direct drug delivery methods, transient disruption of the blood–brain barrier, and stem cell therapies [33,34,35,36,37]. However, none of the treatments have been able to overcome the current challenges fully [32].

## 9. Concluding Remarks and Open Questions

The prion-like strain behavior of α-Syn is still an enigmatic phenomenon. It is surprising how a single protein without any proper structure can fold in many different ways and adopt conformations that result in various pathologies. Many unanswered questions need further investigation, even after an impressive amount of work on α-Syn polymorphs and strains. For instance, what drives the formation of α-Syn strain under given cellular and environmental conditions? How do these strains target different cell types and brain regions? Do α-Syn strains evolve, change, or adapt with time depending upon the host factors? How does the presence of other proteins, ligands, membranes, or co-factors influence strain formation? Do α-Syn strains have the ability to cross-seed with each other and result in mixed pathologies? Do α-Syn strains interfere and block the propagation of each other, similarly to prions? Is it possible, or do we have techniques sensitive enough to discriminate between PD and MSA strains at an early stage of diagnosis? Considering the expansion of the prion strain phenomenon to several other amyloidogenic proteins, it may also be necessary to understand the key molecular events in strain biology and design effective strategies.

## Data Availability

Not applicable.

## Abbreviations

α-synuclein (α-Syn), Parkinson’s disease (PD), multiple system atrophy (MSA), dementia with Lewy body (DLB), Parkinson’s Disease Dementia (PDD), Lewy bodies (LBs), Lewy neurites (LNs), Alzheimer’s disease (AD), Glial cytoplasmic inclusions (GCIs), post-translational modifications (PTMs), wild-type (WT), cryo-electron microscopy (cryo-EM), solid-state NMR (ssNMR) spectroscopy; transmission electron microscopy (TEM), atomic force microscopy (AFM), circular dichroism (CD), dorsal motor nucleus of the vagus nerve (DMV), enteric nervous system (ENS); β-Synuclein (β-syn); γ-synuclein (γ-Syn); Protein misfolding cyclic amplification (PMCA); Liquid-Liquid phase separation (LLPS).
